# Physical Instability and Functional Deterioration of High-Protein Dairy Powders: Mechanisms of Caking, Agglomeration, and Rehydration Loss

**DOI:** 10.3390/molecules31132230

**Published:** 2026-06-24

**Authors:** Marek Szołtysik, Nesa Dibagar, Monika Słupska, Małgorzata Serowik, Artur Gryszkin, Adam Figiel

**Affiliations:** 1 Department of Functional Food Products Development, Faculty of Biotechnology and Food Science, Wrocław University of Environmental and Life Sciences, 37 Chełmońskiego Str., 51-630 Wroclaw, Poland; marek.szoltysik@upwr.edu.pl; 2 Department of Food Chemistry and Biocatalysis, Faculty of Biotechnology and Food Science, Wrocław University of Environmental and Life Sciences, 25 Norwida Str., 50-375 Wroclaw, Poland; 3 Department of Engineering Fundamentals, Institute of Agricultural Engineering, Wrocław University of Environmental and Life Sciences, 37 Chełmońskiego Str., 51-630 Wroclaw, Poland; monika.slupska@upwr.edu.pl; 4 Institute of Agricultural Engineering, Wroclaw University of Environmental and Life Sciences, 37a Chełmońskiego Str., 51-630 Wroclaw, Poland; malgorzata.serowik@upwr.edu.pl (M.S.); adam.figiel@upwr.edu.pl (A.F.); 5 Department of Food Storage and Technology, Faculty of Biotechnology and Food Science, Wrocław University of Environmental and Life Sciences, 37 Chełmońskiego Str., 51-630 Wroclaw, Poland; artur.gryszkin@upwr.edu.pl

**Keywords:** dairy powders, protein, stickiness, caking, glass transition, rehydration, stability

## Abstract

The rapid expansion of high-protein dairy-based powders (HPDPs), including milk protein concentrates and isolates (MPC/MPI), whey protein concentrates and isolates (WPC/WPI), and micellar casein concentrates and isolates (MCC/MCI), has intensified the need to understand instability phenomena that emerge during processing and storage. These products are governed by protein-rich amorphous matrices, in which molecular mobility, interfacial composition, and mineral interactions dictate both physical stability and functional performance. Importantly, these physical instabilities are directly coupled with functional deterioration, particularly in terms of impaired wetting, dispersion, and dissolution during rehydration. This review presents an integrated mechanistic framework linking these instability phenomena across processing, storage, and reconstitution, thereby consolidating concepts that remain fragmented across the current literature on high-protein dairy matrices. Key controlling factors include glass transition temperature (Tg), water activity-induced plasticization, protein–protein and protein–mineral interactions, and surface compositional heterogeneity established during spray drying. These factors govern the progression from surface stickiness through uncontrolled agglomeration to caking, forming a consolidation continuum. In contrast to lactose-driven matrices, caking and agglomeration in HPDPs arise primarily from protein-mediated restructuring and inter-particle bonding, with lactose crystallization acting only as a secondary mechanism in mixed-composition grades. The review further distinguishes engineered agglomeration from storage-induced consolidation and evaluates advances in molecular mobility characterization and Tg-based stability mapping. Significant gaps remain in linking localized surface evolution, mineral redistribution, and inter-particle bridge chemistry under realistic environmental conditions. The review concludes by proposing a mobility-centered “stability-by-design” framework that integrates composition, processing, particle architecture, and storage conditions to guide the development of future HPDPs with improved physical stability and functional recovery.

## 1. Introduction

High-protein dairy-based ingredients have attracted considerable attention in modern food formulation, driven by the rapid expansion of protein-enriched products across sectors including sports nutrition, clinical nutrition, infant nutrition, and functional foods [1,2]. Ingredients such as milk protein concentrates (MPC), milk protein isolates (MPI), micellar casein concentrates (MCC), micellar casein isolates (MCI), whey protein concentrates (WPC), whey protein isolates (WPI), and selected protein-rich dairy co-products such as buttermilk-derived ingredients provide not only high nutritional value but also a wide range of functional properties [3,4,5]. As protein content increases, often exceeding 80–90% in commercial powders, these materials transition into a distinct physicochemical regime characterized by reduced lactose content, altered mineral balance, and increasingly dominant protein–protein interactions that strongly influence powder functionality and stability [6,7].

High-protein dairy-based powders (HPDPs) are typically produced through membrane-based fractionation processes, such as ultrafiltration and diafiltration, followed by spray drying [8,9,10]. These processes enable selective enrichment of proteins while removing lactose and soluble minerals, resulting in powders with tailored composition and functionality. Spray drying subsequently transforms concentrated liquid feed into low-moisture powders by rapid water removal, kinetically trapping components within a predominantly amorphous matrix. However, this transformation also generates metastable structures that are inherently susceptible to physical and functional instability during both processing and storage [11,12].

From an industrial perspective, these instability phenomena manifest as recurring and costly processing and product quality challenges. High-protein milk powders, particularly MPC80 and related MPC grades, are susceptible to caking and clumping during storage at elevated temperatures and humidity, primarily due to moisture-induced plasticization of protein-rich amorphous domains, protein aggregation, and inter-particle bonding, which collectively impair flowability and functional performance [13,14]. Residual lactose crystallization may act as a secondary contributing mechanism in transitional protein grades where lactose content remains significant. Similarly, WPIs stored at elevated temperatures have been shown to develop increased surface hydrophobicity and reduced wettability, directly impairing reconstitution performance [15,16]. Comparable instability phenomena have also been reported across other high-protein dairy powders, including MCC and related protein-rich powders, indicating that these challenges extend beyond individual product classes. These practical observations highlight the need to connect mechanistic understanding with real-world instability scenarios.

In practice, HPDPs frequently exhibit stickiness during drying, caking and uncontrolled agglomeration during storage, reduced flowability under moderate humidity, and progressive deterioration of rehydration performance. Throughout this review, agglomeration refers to storage-induced, uncontrolled particle clustering unless otherwise specified, thereby distinguishing it from intentionally engineered agglomeration processes used for powder instantization [6,12,17]. These phenomena are often treated as separate quality defects; however, they originate from a common underlying mechanism: mobility-driven structural evolution within an amorphous, heterogeneous matrix. Such instabilities reduce process efficiency, increase fouling and product loss, complicate handling and transport, and limit shelf life. Although chemical reactions such as Maillard-induced protein modification, lactosylation, and oxidation may also contribute to quality deterioration, the present review focuses primarily on physical instability mechanisms governed by molecular mobility and structural evolution.

Immediately after spray drying, HPDPs exist predominantly in a glassy state in which molecular mobility is restricted but not eliminated. The stability of this state is strongly governed by the relationship between storage temperature (T) and the glass transition temperature (Tg), as well as by water activity (*a_w_*), which acts as a powerful plasticizer [18,19]. When T approaches or exceeds Tg, molecular mobility increases sharply, enabling viscous flow, structural relaxation, diffusion, and inter-particle bonding. Moisture sorption further depresses Tg and accelerates these processes, making environmental humidity a critical determinant of powder stability [20].

In lactose-rich dairy powders, instability is typically governed by moisture-induced plasticization followed by lactose crystallization, which reinforces inter-particle bridges and accelerates consolidation [6,19]. In fat-containing systems, partial melting and recrystallization of lipids contribute to cohesion through temperature-dependent fat bridging [21]. In contrast, HPDPs represent a fundamentally different regime in which instability is dominated by protein–protein interactions, mineral-mediated associations, and structural densification rather than carbohydrate crystallization. The reduction in lactose and modifications to mineral composition arising from fractionation diminish classical sugar-driven stabilization mechanisms and amplify protein-controlled pathways [6,13,22,23]. These lactose-driven mechanisms are discussed here primarily as a mechanistic reference point, as instability in HPDPs follows fundamentally different pathways dominated by protein-rich amorphous domains and protein–mineral interactions.

**Figure 1:**
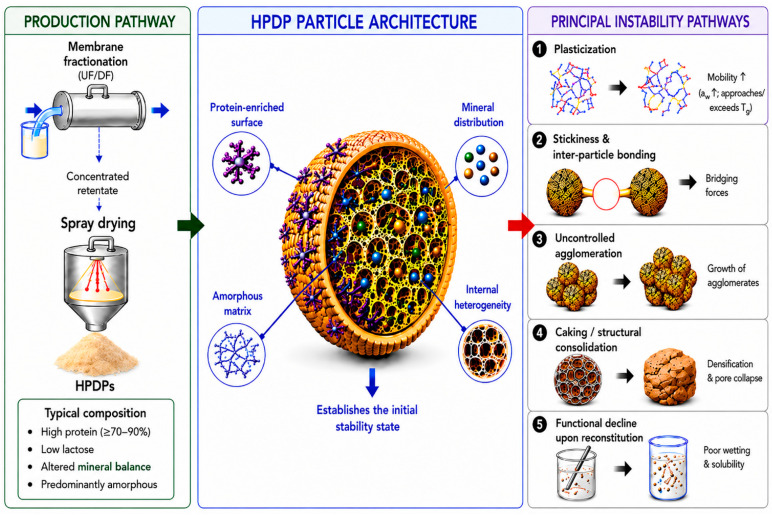
Fundamental particle architecture and instability pathways of HPDPs. UF: ultrafiltration, DF: diafiltration.

The key structural features and instability pathways characteristic of HPDPs are summarized in Figure 1.

Surface composition and particle architecture further play a critical role in determining instability behavior. During spray drying, differential migration of components leads to compositional heterogeneity, often resulting in protein-enriched particle surfaces and internal gradients in density and porosity. These surface layers govern inter-particle adhesion during storage and wetting behavior during reconstitution [12,24]. In many HPDPs, particularly those containing high proportions of surface-active proteins, protein-enriched particle surfaces may exhibit increased surface hydrophobicity, which delays water penetration and contributes to poor wettability [7]. Rehydration of HPDPs is a complex, multistep process involving wetting, penetration, dispersion, and dissolution [25], and each of these stages may be compromised by storage-induced structural changes. Importantly, functional deterioration may be only partially reversible under conventional reconstitution conditions, meaning that physical instability developed during storage can directly and persistently influence functional performance during reconstitution.

Despite extensive research on individual aspects of dairy powder behavior, including storage stability, powder physical properties, and rehydration performance, these topics are frequently addressed independently, often with a focus on conventional lactose-rich dairy powders or specific product classes. However, growing evidence indicates that processing history, storage-induced structural evolution, and rehydration behavior are intrinsically linked in HPDPs, where protein-dominated matrices follow instability pathways that differ fundamentally from those of conventional dairy powders. While previous reviews have addressed dairy powder stability, rehydration, or drying behavior separately, a unified framework linking processing-induced particle architecture, storage-driven instability, and functional deterioration in protein-dominant dairy powders remains lacking. Processing conditions define particle architecture, surface composition, and molecular organization, thereby establishing the initial mobility landscape of the powder [24,26]. Storage conditions modulate this mobility landscape through environmental drivers such as temperature and humidity, leading to progressive structural evolution [21]. Rehydration performance ultimately reflects the cumulative outcome of these transformations [27,28]. A comprehensive understanding of the behavior of HPDPs therefore requires an integrated, cross-scale framework linking composition, processing, structure, molecular mobility, and functional performance.

Recent analytical advances, including dynamic vapor sorption, terahertz spectroscopy, and dynamic mechanical analysis, have significantly improved the ability to probe molecular mobility and structural evolution in amorphous systems [29,30,31]. Nevertheless, their integration into predictive stability frameworks for HPDPs remains limited. In particular, the coupling among localized surface evolution, mineral–protein rearrangements, and inter-particle bridge formation under realistic humidity and temperature cycling conditions is not yet fully understood.

Given the increasing industrial importance of HPDPs and their distinct instability pathways, there is a need for a unified mechanistic framework that explicitly connects physical instability with functional degradation. The present review addresses this need by synthesizing current knowledge on stickiness, caking, uncontrolled agglomeration, and rehydration loss in HPDPs. By integrating molecular mobility concepts and thermomechanical theory with empirical evidence, it proposes a mobility-centered perspective and outlines pathways toward stability-by-design strategies for next-generation dairy ingredients.

## 2. High-Protein Dairy-Based Powders

HPDPs constitute a technologically diverse class of ingredients characterized by complex compositional, structural, and interfacial heterogeneity arising from integrated processing operations. Although these powders are often classified based on protein content, compositional thresholds alone are insufficient to fully describe their physicochemical behavior or predict their susceptibility to instability phenomena.

From a stability perspective, HPDPs are non-equilibrium, protein-dominant amorphous systems in which molecular mobility, interfacial composition, and particle architecture collectively govern both physical stability and functional performance. Powder behavior is therefore determined not only by bulk composition but also by processing history, mineral balance, surface heterogeneity, and internal microstructure [7,12,22].

Unlike conventional lactose-rich dairy powders, such as skim milk powder and sweet whey powder, HPDPs operate within a distinct structural regime in which instability is governed primarily by protein–protein interactions, protein–mineral associations, and mobility-driven matrix restructuring rather than lactose crystallization [22,32]. The progressive removal of lactose during fractionation reduces the contribution of amorphous lactose to glass formation and shifts the dominant stability mechanisms toward protein-controlled molecular mobility, protein association, and mineral-mediated interactions. As a result, moisture sorption in HPDPs predominantly plasticizes protein-rich domains, enabling structural relaxation, inter-particle bonding, and progressive densification even in the absence of significant lactose crystallization [6,19].

This distinction is critical because it shifts the interpretation of instability from discrete phase transitions to a continuous mobility-driven progression. In protein-dominant matrices, consolidation arises through the gradual development of cohesive inter-particle contacts facilitated by protein association and mineral bridging, particularly via colloidal calcium phosphate (CCP)-mediated bridging in casein-rich matrices, as well as surface restructuring. These processes increase the mechanical strength of particle assemblies and reduce porosity, which in turn diminishes accessibility during rehydration. Thus, physical instability phenomena such as uncontrolled agglomeration and caking, including progressive consolidation are directly linked with functional degradation, particularly reduced wettability, delayed dispersion, and incomplete dissolution [13,33].

Importantly, surface properties play a decisive role in governing cohesion during storage and hydration behavior during reconstitution, making particle interfaces critical determinants of both physical stability and functional performance. During spray drying, compositional segregation results in heterogeneous particle structures, typically characterized by protein-enriched surfaces and gradients between the surface and core. These surface layers determine water sorption kinetics, adhesion behavior, and the initiation of inter-particle bonding under humid conditions. At the same time, they may act as barriers to water penetration during rehydration, making surface evolution a key determinant of functional performance [7,28,33].

The susceptibility of HPDPs to moisture-induced instability reflects the plasticizing effect of water on protein-rich amorphous domains, a mechanism distinct from lactose crystallization-driven consolidation in conventional dairy powders, as detailed in Section 1. Consequently, high-protein powders such as MPC80, MPI, MCC, and WPI may exhibit progressive stickiness, uncontrolled agglomeration, caking, and severe rehydration impairment even at relatively low residual lactose levels [6,17,22].

### Structural Classification of HPDPs Based on Origin and Protein Content

HPDPs originate from distinct dairy streams subjected to targeted fractionation and concentration prior to dehydration. These upstream separations define not only the internal compositional architecture of the final particles but also their protein concentration, residual lactose content, mineral distribution, and consequently, their stability behavior. MPCs are typically classified according to protein content on a dry basis, encompassing commercially available grades ranging from approximately 40% to 85% protein. These powders are commonly grouped into low-protein (40–60%), medium-protein (60–70%), and high-protein (≥80%) categories, reflecting compositional differences that influence their physicochemical and functional properties [7,34]. MPIs contain ≥90% protein on a dry basis and represents a further purified form with minimal residual lactose and minerals [3,7,34]. Both are produced from skim milk via ultrafiltration and diafiltration, processes that retain caseins and whey proteins in proportions similar to those in native milk while progressively removing lactose and soluble minerals as protein concentration increases. As protein enrichment advances, the reduction in lactose decreases the fraction of amorphous carbohydrates contributing to the glassy matrix. Consequently, the role of lactose-driven vitrification diminishes, and the matrix becomes increasingly governed by protein–protein and protein–mineral interactions, which alter cohesion mechanisms and moisture sensitivity [3,6].

MCCs typically contain 60–85% protein, while MCIs exceed 90% protein on a dry basis. These powders are obtained through microfiltration of skim milk, which selectively removes whey proteins while preserving intact casein micelles and their associated CCP. The resulting powders are structurally dominated by mineral-stabilized casein aggregates with high protein density and strong CCP-mediated associations. Elevated protein and mineral contents promote dense inter-particle contact zones, which may increase susceptibility to cohesion and reduced rehydration performance under humid storage conditions [34,35].

WPCs are commercially available across a broad range, typically from 35% to 80% protein, whereas WPIs generally contain ≥90% protein on a dry basis [7,34]. These ingredients are derived from cheese whey using ultrafiltration and diafiltration. In some cases, ion-exchange chromatography is employed as an alternative purification route, producing WPI fractions selectively enriched in specific whey proteins, notably β-lactoglobulin. At higher protein grades, residual lactose becomes minimal or negligible. The predominance of globular whey proteins, combined with reduced carbohydrate content, enhances interfacial activity and sensitivity to moisture-induced structural rearrangements. High-protein WPIs, in particular, may exhibit aggregation, surface stickiness, and consolidation driven by localized plasticization and protein–protein bonding [7,12,22].

Buttermilk powder (BMP) typically contains moderate protein levels of approximately 30–40%, depending on processing conditions and the efficiency of fat separation during butter manufacture [36]. Although BMP does not meet the conventional high-protein threshold applied to MPCs, WPIs, or MCCs, it is discussed here as a mechanistically informative comparator rather than as a representative HPDP. Its relevance arises from the role of milk fat globule membrane (MFGM) components, including phospholipids, membrane proteins, and residual caseins and whey proteins, in governing particle surface composition, inter-particle adhesion, and wettability [36]. The presence of phospholipid-rich interfaces and residual fat may promote surface mobility and structural heterogeneity during storage, particularly under conditions of elevated temperature and humidity, providing a useful mechanistic contrast to purely protein-dominated systems [21,36]. Buttermilk protein concentrate (BMPC), produced by further ultrafiltration of buttermilk, can reach 60–80% protein and exhibits instability behavior more closely aligned with conventional HPDPs; however, its study remains limited in the published literature.

**Figure 2:**
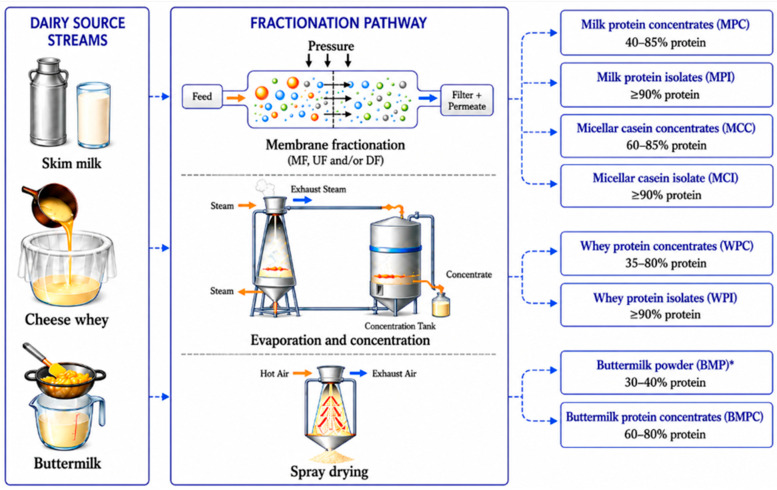
Classification of selected dairy protein powders according to source, fractionation pathway, and protein content. * Included as a mechanistically relevant comparator despite not meeting conventional HPDP protein thresholds.

Figure 2 schematically represents selected HPDPs in relation to their dairy source, fractionation pathway, and resulting protein content. The diversity of compositional and structural architectures across these HPDP classes gives rise to distinct instability pathways that cannot be predicted from protein content alone. Surface composition, mineral balance, and processing history collectively define the molecular mobility landscape of each powder type, thereby determining its susceptibility to stickiness, uncontrolled agglomeration, caking, and rehydration loss. The mechanistic underpinnings of these phenomena are examined in detail in Section 3.

## 3. Mechanistic Definitions of Dominant Physical Instabilities in HPDPs

Understanding the behavior of HPDPs requires a mechanistic framework that moves beyond descriptive classification of defects toward a process-based interpretation of instability development. Building on the compositional and structural framework established in Section 2, the following subsections define and mechanistically interpret the dominant physical and functional instabilities observed in HPDPs, namely stickiness, uncontrolled agglomeration, caking, and loss of rehydration performance. These phenomena are not treated here as fully independent instability classes but as operationally distinguishable stages within a common mobility-driven cohesion–aggregation–consolidation continuum.

Within this framework, stickiness represents the onset of adhesive interactions caused by surface plasticization and reduced viscosity; uncontrolled agglomeration represents the intermediate stage in which multiple adhesive contacts organize into discrete particle clusters; and caking represents the advanced stage in which inter-particle bridges strengthen into mechanically resistant structures [18,19,21,31,33]. Loss of rehydration performance represents the functional consequence of these structural and interfacial transformations, although it may also arise before severe macroscopic caking becomes visible [22,25,27]. Since HPDPs are structurally heterogeneous matrices, these stages may overlap or coexist depending on local water activity, temperature, packing density, particle surface composition, and storage history [21,26,32]. Therefore, the distinction among stickiness, uncontrolled agglomeration, and caking is mechanistic and operational rather than absolute.

A further distinction must be made between thermodynamic state descriptors and kinetically controlled instability processes. Tg, *a_w_*, moisture sorption, and the difference between T and Tg (ΔT) describe the physical state of the powder matrix and its potential for molecular mobility. However, the visible instability outcomes, including adhesion, agglomerate growth, bridge strengthening, caking, and rehydration loss, are time-dependent kinetic processes. Thus, exceeding a critical mobility threshold does not instantaneously produce caking or insolubility; rather, it increases the rate at which structural relaxation, viscous flow, inter-particle bonding, and consolidation proceed. This distinction is essential because HPDP stability depends not only on whether the powder is in a mobility-favorable state but also on exposure time, local humidity gradients, packing stress, particle architecture, and surface heterogeneity [14,37].

Instability in HPDPs is fundamentally influenced by the relationship between T and Tg and by moisture-induced plasticization, which together determine the mobility potential of the amorphous or protein-rich matrix. Once sufficient mobility is achieved, time-dependent processes such as surface relaxation, viscous flow, inter-particle bridge formation, and structural consolidation may proceed [13]. As these transformations progress, they alter bulk powder properties, such as flowability, porosity, and mechanical strength, while also creating barriers to water penetration during rehydration. This directly reinforces the coupling between physical instability and functional degradation in HPDPs [13,37].

### 3.1. Stickiness: Onset of Mobility-Enabled Adhesion

Stickiness represents the earliest stage of cohesion development in HPDPs and manifests both during manufacturing and storage as a consequence of moisture-activated molecular mobility at particle surfaces [38]. In the context of the storage-instability continuum, stickiness corresponds to the initial formation of adhesive contacts between particles. These interactions are generally weak, localized, and at least partially reversible, particularly before extensive bridge strengthening or structural consolidation occurs [39].

The term stickiness is used in two related but operationally distinct contexts. During spray drying, stickiness refers mainly to the adhesion of partially dried droplets or particles to dryer walls or other particles when the particle surface temperature approaches or exceeds the effective Tg of the amorphous surface matrix [18,19]. Under these conditions, the matrix enters a high-mobility state and surface viscosity decrease, increasing the probability of adhesion during collision events [24]. Wall deposition is often practically irreversible within the drying process and reduces powder yield while contributing to fouling, particularly in high-protein systems that exhibit narrow operating windows between outlet air temperature and the effective matrix Tg [24,40].

During storage, stickiness refers primarily to particle–particle adhesion within the powder bed. Moisture uptake plasticizes particle surfaces, lowers local viscosity, and promotes transient liquid or highly viscous bridges between adjacent particles, especially at contact points subjected to compressive stresses [18,19,21,31,41]. Unlike caking, which involves the development of mechanically stable solid bridges, early-stage storage stickiness is characterized by reduced surface viscosity and weak inter-particle interactions. However, these adhesive contacts provide the structural foundation for subsequent uncontrolled agglomeration and caking if mobility persists over time. The extent and persistence of stickiness depend on water activity, temperature, contact time, matrix composition, surface composition, and packing conditions [13,19,41].

In HPDPs, stickiness is primarily governed by protein-driven plasticization and surface restructuring rather than by lactose crystallization alone. Protein-rich amorphous domains exhibit moisture-dependent softening and structural relaxation, which may occur even at relatively low lactose levels. As a result, powders such as WPI and high-protein MPC may display significant surface stickiness despite the absence of dominant lactose crystallization phenomena [15,33]. Surface composition plays a decisive role in this behavior. Protein-enriched surfaces, formed through compositional segregation during spray drying, influence hygroscopicity, surface energy, and local mobility. These heterogeneous surface layers may undergo localized plasticization, creating regions of reduced viscosity that act as initiation sites for adhesion and inter-particle contact formation [13,32]. This surface-controlled behavior is particularly critical in HPDPs, where interfacial properties govern early-stage cohesion during storage and strongly affect subsequent rehydration performance.

### 3.2. Uncontrolled Agglomeration: Intermediate Cluster Formation Between Stickiness and Caking

Within the cohesion–consolidation continuum, uncontrolled agglomeration represents the intermediate stage between early stickiness and advanced caking. It occurs when multiple adhesive particle–particle contacts, initially formed through surface plasticization and reduced viscosity, organize into discrete clusters before the system develops the continuous mechanical strength characteristic of caking. Thus, uncontrolled agglomeration should not be interpreted as an instability class fully independent of stickiness or caking but rather as a transitional structural state within the mobility-driven evolution of HPDPs.

Agglomeration in HPDPs must also be distinguished by origin and timing, as similar macroscopic structures may arise from either controlled processing operations or storage-induced instability. Engineered agglomeration, often referred to as instantization, is intentionally applied after drying through controlled granulation processes. In this case, binder addition, particle collision, and subsequent drying generate large, porous granules designed to enhance capillary water uptake and improve wettability and, in some cases, dispersibility [33,40,42]. However, evidence from HPDPs indicates that engineered agglomeration does not necessarily improve solubilization in micellar casein-dominant powders because internal protein–protein associations may remain the primary limitation to dissolution. In such systems, binder selection may improve wetting behavior without significantly altering dispersion or dissolution kinetics [22,25].

In contrast, uncontrolled agglomeration develops during storage as a consequence of moisture-induced plasticization, increased molecular mobility, and the persistence of adhesive inter-particle contacts at contact points. As relative humidity (RH) and/or temperature (T) increase, surface viscosity decreases, promoting adhesion and the formation of multiple inter-particle contacts. This process is closely related to stickiness but represents a more advanced stage of structural organization, where initially localized adhesive interactions extend into particle clusters [21,40].

In HPDPs, uncontrolled agglomeration is primarily governed by protein-driven mechanisms, including surface plasticization, protein–protein interactions, and mineral-mediated bridging. Early stage agglomerates may remain partially reversible; however, continued exposure to conditions that increase molecular mobility promotes capillary bridge development, viscous flow or sintering-like contact-zone deformation, and progressive strengthening of inter-particle bonds. Under more severe or prolonged storage conditions, this process can evolve into caking, where inter-particle bridges progressively strengthen into mechanically resistant solid connections. This progression has been reported even in low-lactose systems such as WPI and caseinate powders, confirming that uncontrolled agglomeration and caking can develop through protein-dominant mechanisms rather than lactose crystallization alone [5,13,14].

Functionally, uncontrolled agglomeration becomes critical when cluster formation disrupts powder flowability, reduces dosing uniformity, and impairs hydration performance by limiting particle dispersion and water penetration [40]. Its significance therefore lies not only in the formation of visible clusters but also in representing the transition from reversible adhesive interactions toward progressively irreversible structural consolidation.

**Figure 3:**
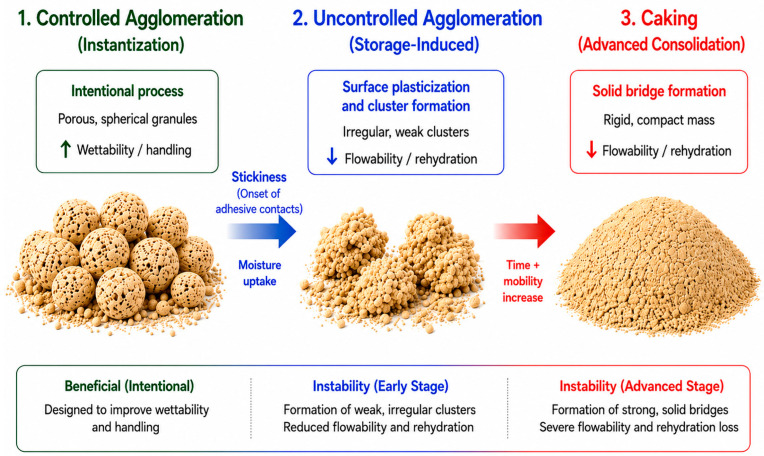
Distinction and relationship between controlled agglomeration, uncontrolled agglomeration, and caking in HPDPs.

Figure 3 illustrates three structurally related but operationally distinguishable powder states within the broader cohesion–consolidation framework.

Controlled agglomeration is an intentional post-drying process that produces large, porous granules to improve wettability and handling [33,40]. In contrast, uncontrolled agglomeration arises during storage as a result of moisture sorption, surface plasticization, and mobility-enabled particle adhesion, leading to the formation of irregular, weakly to moderately bound clusters with reduced flowability and inconsistent rehydration [18,19,21,40]. With continued exposure to moisture and time, these agglomerates may undergo consolidation through inter-particle bridge formation and strengthening, ultimately resulting in caking, a rigid and mechanically stable state characterized by severe loss of flowability and poor rehydration performance [21,42]. The figure emphasizes that controlled agglomeration is a beneficial processing strategy, whereas uncontrolled agglomeration and caking represent progressive stages of instability driven by mobility-mediated structural transformation [33,40,42].

### 3.3. Caking: Advanced Consolidation and Bridge Strengthening

Caking represents the advanced consolidation stage within the mobility-driven instability continuum [21,42]. It develops when initially adhesive or weakly agglomerated particles progressively acquire cohesive strength through inter-particle bridge formation and subsequent bridge strengthening [21,42]. In contrast to stickiness, which involves weak and often partially reversible adhesive interactions [39], caking is characterized by the formation of mechanically resistant structures and substantial loss of powder flowability [21,42].

The progression toward caking typically begins with moisture sorption at particle surfaces and contact points, where water acts as a plasticizer that depresses Tg, lowers viscosity, and enables molecular mobility [18,19,21]. Once the powder matrix reaches a mobility-favorable state, kinetic processes such as viscous flow, capillary condensation, structural relaxation, and inter-particle bond formation proceed over time [13,14,21,42]. In the early stages, capillary and viscous forces promote transient liquid or highly viscous bridges between neighboring particles, particularly under the compressive stresses present in packed powder beds [42]. With increasing exposure time and sustained molecular mobility, these bridges strengthen through viscous flow, sintering-like contact-zone deformation, structural relaxation, and, in protein-rich systems, protein network consolidation [13,14,21,42]. This ultimately results in the formation of mechanically resistant solid bridges and loss of flowability [13,14,21,42]. As cohesive structures develop, the associated reduction in porosity and increase in mechanical strength significantly impair water penetration during rehydration, contributing to delayed dispersion and incomplete dissolution [13,37,43].

In high-protein, low-lactose HPDPs, caking is usually governed more strongly by protein-driven plasticization, protein–protein interactions, and mineral-mediated bridging than by lactose crystallization [13,14,37]. Due to the reduced lactose content and increased dominance of protein-rich phases, consolidation occurs mainly through moisture-induced plasticization, structural relaxation, interfacial rearrangement, and the formation of cohesive inter-particle bonds [13,14,21]. In casein-rich systems, mineral-mediated associations and micellar restructuring may further contribute to contact-zone consolidation [13,37,43]. These processes typically result in gradual and progressive strengthening, reflecting continuous mobility-driven structural evolution within protein-dominant matrices [13,14,21,37].

In lactose-rich dairy powders, by contrast, lactose crystallization may act as an additional consolidation mechanism [18,19,44]. Crystallization is not merely a downstream consequence of moisture uptake; it can actively modify powder microstructure, surface chemistry, and reconstitution behavior by transforming amorphous lactose into crystalline forms under humidity-dependent storage conditions [44,45,46]. This transition is associated with increased molecular mobility above Tg and proceeds at rates that depend strongly on storage conditions such as RH [18,19,45,46]. As lactose crystallization progresses, changes in the distribution and physical state of water within the powder matrix may intensify local mobility and promote the transition from weak, reversible cohesion to stronger, less reversible inter-particle bonding and caking, particularly in lactose-rich systems [18,19,44,45,46].

Empirical storage studies of lactose-containing whey powders support this mechanism, showing that caking is strongly associated with intermediate water activity conditions where sorption isotherms often exhibit discontinuities consistent with amorphous-to-crystalline lactose transitions. In such systems, caking develops once powder water activity enters a practical threshold window of around 0.3–0.4 at ambient temperature, consistent with plasticization followed by crystallization-driven bridge reinforcement [47,48]. Although this behavior is not fully representative of HPDPs, analogous behavior has been reported for whole milk powder under high-humidity storage, where moisture content initially increases and subsequently decreases following lactose crystallization, illustrating how crystallization-driven moisture redistribution can contribute to localized consolidation [49]. Low-field NMR and related mobility-sensitive analyses have further reinforced the practical relevance of this boundary by detecting lactose crystallization signatures at water activities above approximately 0.4, supporting the view that this range represents a meaningful stability threshold in lactose-containing systems [47].

Although permeate powders are not representative HPDPs, they provide a useful contrast model for understanding crystallization-driven caking in lactose-dominant matrices. Whey permeate and deproteinized whey-derived permeate powders are intrinsically lactose rich and therefore occupy the most crystallization-sensitive region of the dairy powder stability spectrum [46,50]. Industrial manufacture typically involves concentration of permeate, controlled lactose crystallization prior to spray drying, drying of a slurry containing lactose crystals, followed by post-crystallization and final drying [50]. This process is designed to reduce the fraction of amorphous lactose and improve handling stability [46,50].

This strategy reflects a well-established principle in dairy powder technology: crystalline lactose is relatively stable at high relative humidity compared with amorphous lactose [6,46,51], and maximizing lactose crystallinity is commonly used to reduce stickiness and caking during drying, transport, and storage [46,50,51]. Nevertheless, permeate powders may remain sensitive in practice because crystallization concentrates non-lactose components, including salts and residual nitrogenous compounds, into a continuous amorphous phase that often exhibits a low Tg and a strong moisture plasticization response [52]. Recent structure–property analyses have proposed a two-phase model in which lactose crystals are embedded within or coated by an amorphous matrix enriched in non-lactose components; this amorphous fraction controls water sorption, plasticization, and the initiation of caking at particle–particle contacts despite high overall crystallinity [52].

Consistent with this view, crystallization kinetics studies comparing amorphous lactose, whey powder, and whey permeate powders show that increasing temperature and relative humidity significantly accelerate the combined sorption–induction–crystallization process, indicating that transient environmental fluctuations can rapidly induce crystallization-coupled bridge strengthening even in partially crystalline powders [45,52]. X-ray diffraction studies further demonstrate that the polymorphic form of lactose depends on temperature and humidity history, with α-lactose monohydrate typically forming under storage-relevant conditions, while less stable anhydrous forms may appear at elevated temperatures, potentially influencing subsequent stability behavior [53].

**Figure 4:**
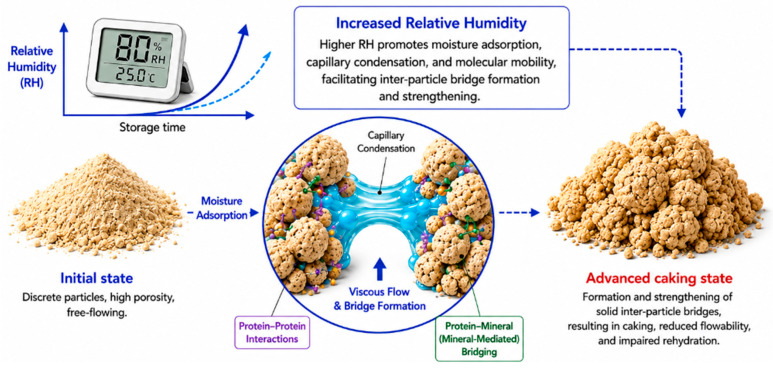
Mechanistic representation of caking development in HPDPs under elevated RH.

Figure 4 schematically represents the advanced consolidation stage of HPDP instability, in which prior adhesive contacts and weak agglomerates evolve into mechanically resistant caked structures under elevated relative humidity and temperature. The macroscopic progression illustrates the transition from a free-flowing powder to a consolidated caked structure as a result of moisture adsorption and thermally enhanced molecular mobility. The upper panel highlights the influence of increasing storage severity relative to critical mobility thresholds. The magnified inset depicts the particle-scale mechanisms underlying caking, including capillary condensation, viscous flow, and the formation of solid, resistant inter-particle bridges driven by protein–protein and protein–mineral interactions. This multiscale framework emphasizes the coupling between environmental conditions, molecular mobility, and time-dependent structural evolution governing physical instability in high-protein powders.

### 3.4. Insolubility: Loss of Rehydration Performance

Insolubility, more precisely described in HPDPs as loss of rehydration performance, represents the functional consequence of progressive physical instability and manifests during reconstitution, although it commonly originates from structural and interfacial evolution during storage [25,37]. Within the mobility-driven framework described above, it can be interpreted as the end-stage outcome of sequential transformations from stickiness to uncontrolled agglomeration and ultimately consolidation [21,37,42]. It is mechanistically heterogeneous and should not be interpreted as purely chemical insolubility, as “poor solubility” measurements often integrate failures in wetting, dispersion/disintegration, and dissolution [28,37].

Loss of apparent solubility may arise from multiple interrelated mechanisms, including impaired wetting due to protein-enriched or chemically modified particle surfaces, delayed dispersion caused by cohesive storage-induced agglomerates, incomplete particle disintegration, and persistent sediment formation under standardized test conditions [25,37]. In HPDPs, particularly casein-micellar-rich powders (MPC80+, MCC/MCI), rehydration loss is frequently linked to mobility-enabled surface and near-surface restructuring, such as crust or skin formation and pore closure, which inhibit water penetration, micellar release, and particle breakup. These processes are typically more critical than bulk irreversible protein denaturation in determining rehydration performance [17,54].

Moisture-driven structural rearrangement, mineral-mediated association, and progressive reduction in effective porosity further hinder water ingress and swelling, slowing the transition from dispersion to complete dissolution and reducing dispersibility and solubility indices [22,34,37]. These effects reflect the surface-controlled nature of rehydration in HPDPs, where interfacial barriers formed during storage limit the accessibility of internal structures [25,37]. A key mechanistic indicator of this behavior is sensitivity to the ionic environment. In high-protein MPC systems, low ionic strength significantly retards rehydration, whereas increased ionic strength and temperature enhance dispersion and dissolution, consistent with mineral-mediated and electrostatic controls on micellar interactions and release [22,55].

Casein-rich powders are particularly prone to mineral-mediated association, surface micelle restructuring and contact-zone consolidation, which may be accompanied by surface lipid migration and increased inter-particle and micelle–micelle interactions that slow rehydration processes [23,54]. In contrast, whey-protein-dominant powders tend to exhibit aggregation state evolution and surface property changes that impair wetting and dispersion. Storage-induced increases in surface hydrophobicity and lactose-dependent aging effects have been demonstrated for WPI under controlled conditions, supporting a coupled physical–chemical pathway to rehydration impairment [15,16].

**Figure 5:**
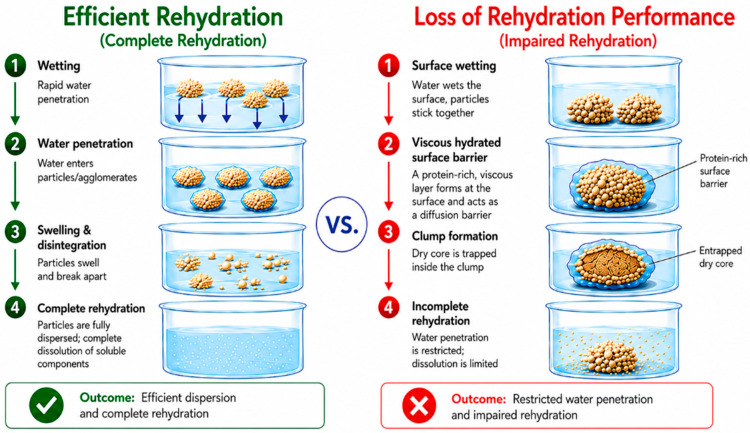
Schematic representation of rehydration behavior in HPDPs, comparing efficient dissolution with rehydration loss (insolubility).

Figure 5 schematically represents rehydration behavior in HPDPs, comparing efficient dissolution with rehydration loss (insolubility). The desirable pathway involves rapid wetting, dispersion, and complete dissolution, whereas the undesirable pathway involves clumping, formation of a surface barrier, and incomplete dissolution due to moisture-induced inter-particle cohesion and restricted water penetration. Rehydration of HPDPs can follow two distinct pathways depending on particle structure, surface properties, and inter-particle interactions. In the undesirable pathway, rehydration loss (insolubility) is initiated when strong cohesion between neighboring particles promotes immediate clump formation upon contact with water. As wetting begins, a gelatinous or highly viscous surface layer rapidly develops at the powder–liquid interface, acting as a diffusion barrier that restricts water penetration into the interior of the agglomerate. Consequently, dry material becomes entrapped within the clump, and dissolution is significantly delayed, occurring only through slow diffusion across the dense outer layer. In contrast, the desirable rehydration pathway is characterized by efficient wetting and rapid penetration of water into individual particles or loosely bound agglomerates. These particles readily sink, absorb water, and undergo swelling, followed by disintegration into smaller units. This progressive dispersion increases the effective surface area available for mass transfer, enabling rapid and complete dissolution. Thus, the transition between these pathways is governed by the balance between inter-particle cohesion and structural accessibility, which ultimately determines rehydration performance in high-protein dairy powders.

### 3.5. Mechanistic Integration of Instability Development in HPDPs

Collectively, the instability phenomena described above represent interconnected manifestations of moisture-enhanced molecular mobility in protein-dominant matrices, occurring across both processing and storage stages [13,14,18,19,21]. Stickiness may emerge during drying and early storage as surface viscosity decreases and transient adhesion develops [24,38,40]. Uncontrolled agglomeration follows when increased molecular mobility promotes the formation of multiple inter-particle contacts and discrete clusters [33,40,42]. Caking reflects advanced consolidation under prolonged or more severe exposure, where inter-particle bridges strengthen into mechanically resistant structures [21,42]. Finally, loss of rehydration performance represents the functional consequence of structural densification, surface evolution, pore closure, and restricted water penetration during reconstitution [25,28,37,43]. These physical and functional instabilities and their associated mechanisms are summarized in Table 1.

From a mechanistic perspective, these transitions are governed by the interplay between environmental conditions, including T, RH, and *a_w_*, and intrinsic material properties, including Tg, composition, surface chemistry, mineral distribution, and particle architecture [13,14,18,19,37,41]. Thermodynamic state variables define whether the powder matrix is in a low- or high-mobility condition, whereas visible instability phenomena develop through kinetic processes [18,19,41]. Water activity, moisture sorption, Tg, and ΔT determine the mobility potential of the matrix [18,19,41]. In contrast, adhesion, agglomerate growth, viscous sintering, bridge strengthening, pore closure, and rehydration failure evolve over time and depend on exposure duration, local humidity gradients, packing stress, and powder heterogeneity [21,37,42,56]. Therefore, Tg depression or a positive ΔT should be interpreted as enabling conditions rather than direct evidence that caking or rehydration loss has already occurred [18,19,37,41,56].

In HPDPs, instability progression is typically continuous and mobility-driven rather than being characterized by abrupt phase transitions [13,14,37,41]. Moisture-induced plasticization, protein–protein interactions, mineral-mediated associations, and surface restructuring are usually more important than carbohydrate crystallization, especially in high-protein, low-lactose powders [13,14,21,37]. However, residual lactose may still contribute to instability in transitional or whey-derived powders, particularly when amorphous lactose plasticization and crystallization interact with protein-rich surface and contact-zone processes [18,19,57,58].

This framework can be illustrated through practical storage scenarios. For example, high-protein MPC80 powders stored at moderate relative humidity may initially exhibit increased surface stickiness due to moisture-induced plasticization [13,14,21]. As storage continues, localized adhesion leads to the formation of small agglomerates, particularly in regions of higher packing density [21,42]. With prolonged exposure or slight increases in humidity or temperature, these agglomerates may evolve into cohesive, mechanically stable structures through viscous sintering and protein-mediated bonding, resulting in caking [13,14,21,42]. Upon reconstitution, such powders frequently display delayed wetting, incomplete dispersion, and persistent sediment, reflecting the cumulative effects of structural densification and surface barrier formation developed during storage [17,22,37].

A similar but not identical progression has been observed in WPIs, where storage at elevated temperature and humidity promotes surface restructuring and increased hydrophobicity. In these systems, early-stage stickiness and agglomeration may not be visually severe but can still significantly impair wettability and dispersion during rehydration [15,16]. This highlights that functional degradation may occur even in the absence of pronounced macroscopic caking, reinforcing the importance of surface-controlled mechanisms in HPDP stability [15,16,25,37].

These examples emphasize that physical instability in HPDPs is not the result of isolated events but rather a continuous, condition-dependent progression governed by molecular mobility, interfacial evolution, and time-dependent structural transformation. Different instability phenomena may overlap or coexist depending on local environmental conditions and powder heterogeneity. Therefore, predictive understanding and control of HPDP stability require an integrated perspective that links processing history, storage conditions, and material structure to both physical behavior and functional performance.

### 3.6. Comparative Classification of HPDPs and Dominant Stability Risks

Building on the mechanistic framework outlined in Section 3.5, HPDPs can be compared according to their origin, fractionation pathway, and resulting structural organization. These upstream processing routes determine not only protein concentration but also the balance between residual lactose, mineral distribution, and the integrity of protein assemblies, all of which collectively govern powder behavior during processing, storage, and rehydration [7,59].

Unlike conventional dairy powders, where instability is largely driven by lactose crystallization and glass transition phenomena, HPDPs operate within a protein-dominated regime in which moisture-induced molecular mobility promotes progressive structural evolution [6,19]. In this context, differences in protein composition (casein- vs. whey-dominated), micellar integrity, and mineral-mediated interactions lead to distinct pathways of cohesion, caking, and rehydration impairment [17,22]. As a result, stability cannot be interpreted solely on the basis of composition but must be understood through the interplay between structure, surface properties, and mobility-driven transformations [25,26]. Table 2 therefore provides a comparative classification of HPDPs based on their origin and fractionation route, dominant compositional and structural control, and the principal stability risks most consistently reported in the literature. This classification highlights how variations in upstream processing translate into distinct instability profiles, offering a system-level perspective that links material design to physical stability and functional performance [15,16]. Caseinates are included for comparative purposes, as they represent HPDPs derived through chemical processing rather than membrane fractionation and therefore exhibit distinct structural and functional behavior.

## 4. Key Drivers of Physical Instability in HPDPs

The susceptibility of HPDPs to physical instability is shaped by two interconnected classes of drivers. The first arises from processing history, which defines the initial structural, compositional, and interfacial state of the powder. The second arises during storage, where environmental conditions determine whether this initial state remains stable or evolves through mobility-driven, time-dependent transformations. In this framework, processing variables establish the initial molecular mobility landscape, whereas storage variables, such as temperature, RH, and *a_w,_* determine the thermodynamic state of the powder matrix and influence the rate at which kinetic instability processes proceed.

This distinction is essential for interpreting physical instability in HPDPs. Thermodynamic state descriptors, including Tg, *a_w_*, moisture sorption, and ΔT, indicate whether the matrix is in a low- or high-mobility condition [18,19,21]. However, visible instability outcomes, including stickiness, uncontrolled agglomeration, caking, surface densification, and rehydration loss, develop through kinetic processes that require time, contact, diffusion, relaxation, and structural rearrangement [13,14,21,42]. Therefore, the drivers discussed in this section should not be interpreted as producing instability instantaneously but rather as controlling the probability and rate of instability development [18,19,21,42].

Case-study evidence across HPDPs illustrates this distinction. In MPC80 powders stored under controlled temperature and RH conditions, casein micelle crosslinking at the particle surface leads to the formation of a dense surface crust that slows dissolution and progressively reduces solubility and dispersibility [67]. This surface restructuring mechanism has been further quantified by monitoring water absorption rates in fresh versus stored MPC 80, 85, and 90 powders at 25 and 40 °C, where differences in water activity uptake were directly attributed to casein crosslink formation at the particle surface [37]. MPC powders containing approximately 70–88% protein exhibited increased cohesiveness and reduced flowability with increasing storage temperature, consistent with time-dependent mobility-driven consolidation [68].

In whey-based matrices, WPC34 and WPC80 powders stored under elevated temperature and RH showed progressive deterioration in handling, flowability, and caking behavior, accompanied by physicochemical changes, including protein denaturation, lactosylation-induced surface modification, and increased particle hydrophobicity [58,69,70]. In WPIs, even small differences in residual lactose content significantly influence aging behavior under elevated-temperature storage, as lactosylation, driven by moisture availability, temperature, and reducing sugar content, increases surface hydrophobicity in ways that impair wettability and rehydration, highlighting the sensitivity of these matrices to compositional variation, surface evolution, and moisture-enabled mobility [15,16,58]. The processing and storage drivers underlying these observations are examined in Section 4.1 and Section 4.2, respectively.

### 4.1. Processing Drivers of Physical Instability

Processing conditions play a fundamental role in defining the structural and interfacial properties of HPDPs, thereby establishing their inherent susceptibility to subsequent instability. Key attributes, including particle morphology, porosity, surface composition, protein association state, mineral balance, residual moisture, and *a_w_*, are determined during membrane fractionation, concentration, and spray drying. These attributes do not necessarily induce instability during processing itself; rather, they define the initial structural state and mobility potential of the powder, which later govern its response to environmental exposure during storage and rehydration.

#### 4.1.1. Membrane Filtration and Pre-Drying Structural Organization

Upstream fractionation processes, including ultrafiltration (UF), diafiltration (DF), and microfiltration (MF), define the initial compositional and colloidal state of HPDP feed concentrates [8,9]. These processes determine protein concentration, residual lactose content, mineral distribution, ionic strength, and the integrity of protein assemblies [7,34,35,64], thereby establishing the structural framework that governs subsequent drying behavior, storage stability, and rehydration performance [13,22].

In protein concentrates and isolates, DF and related mineral management steps modify ionic strength and calcium equilibrium, which in turn influence casein micelle integrity, CCP solubilization, and protein–mineral interactions. These changes directly affect protein association state and interfacial behavior, with important consequences for powder rehydration, cohesion, and consolidation during storage [6,71]. In this context, mineral composition should be interpreted as a structural preconditioning factor that defines interaction potential within the protein matrix. It establishes the interaction potential of the protein matrix, while subsequent humidity and temperature exposure determine whether these interactions evolve into kinetic instability outcomes.

Crowley et al. [22] and Babu and Amamcharla [60] demonstrated that low ionic strength strongly impaired MPC rehydration, whereas increased ionic strength combined with temperature accelerated rehydration. These findings provide direct evidence that upstream mineral removal and retention can dominate HPDP functionality even before drying conditions are considered. The filtration route also modifies protein association behavior and thermal response prior to drying. Coşkun et al. [72] showed that UF concentrates exhibited reduced particle diffusivity and distinct heat-induced structural responses compared with MF-derived systems, attributing these differences to variations in mineral balance and whey protein content. These results confirm that filtration strategy influences colloidal organization and thereby affects downstream processability, storage stability, and rehydration behavior.

At the industrial level, variability in solubility and functional performance among commercial MPC and MPI powders has frequently been attributed to differences in processing history, particularly mineral composition and protein association state. So-called solubility classes of milk protein powders often reflect structural differences established during filtration and concentration rather than intrinsic protein content alone [7,55]. Collectively, these studies demonstrate that membrane filtration is not merely a compositional separation step but a critical structural preconditioning stage that defines the physicochemical framework within which drying, storage, and rehydration processes subsequently occur.

#### 4.1.2. Concentration Level and Feed Properties

The concentration level of dairy protein feeds prior to spray drying exerts a decisive influence on the structural architecture and subsequent physical stability of high-protein powders. Increasing total solids content modifies intermolecular spacing, rheological behavior, and droplet formation dynamics. Together, these modifications shape the microstructure that becomes kinetically fixed in the amorphous state upon spray drying [26,73]. As protein concentration increases during ultrafiltration and evaporation, macromolecular crowding intensifies, reducing intermolecular distances and promoting protein–protein interactions [7,13,22]. In MPCs, elevated total solids are associated with increased viscosity and closer casein micelle spacing, thereby intensifying protein–protein interactions. As protein content rises, lactose-mediated spacing is reduced, and the matrix becomes increasingly protein-dominated [74].

Feed viscosity is a critical consequence of concentration level. As total solids rise, viscosity increases nonlinearly due to protein–protein interactions and mineral-mediated associations [26,75]. High-viscosity feeds influence atomization efficiency, typically producing larger droplets and broader droplet size distributions during spray drying [75]. Larger droplets generate particles with altered drying profiles, thicker surface crusts, and modified internal porosity, directly affecting moisture diffusion pathways, mechanical compliance, and surface composition heterogeneity [24,26,58].

Case studies illustrate these effects clearly. Crowley et al. [22] found that MPC powders produced from higher-solids feeds showed altered morphology and reduced rehydration performance, likely reflecting stronger protein association and pre-drying densification. Zhou et al. [76] reported that higher protein concentration reduced solubility and increased storage-induced aggregation under humid conditions, indicating that concentration-induced packing can be kinetically fixed during drying and subsequently manifest as poor reconstitution.

Concentration level also influences internal moisture gradients during drying [12,24]. Highly concentrated feeds contain less free water relative to solids, promoting rapid surface vitrification and the potential formation of rigid outer shells while the particle core remains relatively plasticized [12,24]. This gradient generates internal stresses and heterogeneous density distributions [24,26]. Upon storage, such heterogeneity may promote localized plasticization at contact points, enhancing the likelihood of inter-particle bonding and caking [42,77].

Mineral balance interacts strongly with concentration effects. In casein-rich systems, increasing concentration elevates the relative proportion of colloidal calcium phosphate within the retentate, reinforcing micellar interactions. Under storage conditions, calcium-mediated bridging can facilitate cohesive contact formation when moisture lowers matrix viscosity [78]. In whey-protein-dominant matrices, high concentration combined with thermal exposure during drying may promote pre-aggregation, reducing dispersibility and accelerating rehydration impairment [58,79].

#### 4.1.3. Spray Drying and Particle Formation

Atomization generates droplets with very high interfacial area, and the subsequent coupled heat and mass transfer promotes selective redistribution of components as the droplet concentrates [12,80]. In high-protein dairy systems, the final particle surface does not necessarily reflect bulk composition [58]. Proteins and lipids may be overrepresented at or near the surface, whereas lactose is often preferentially concentrated toward the particle core [79,81]. This compositional segregation is sensitive to drying conditions, particularly outlet air temperature, outlet RH, drying rate, and the timing of surface skin formation [16,81].

In a study designed to resolve protein localization, Paul et al. [79] demonstrated that whey proteins were strongly enriched at the particle surface, whereas casein micelles remained predominantly in the particle interior. This layered organization was directly linked to sequential swelling and elution behavior during rehydration, confirming that spray-drying-induced stratification has functional consequences. As evaporation proceeds, solutes accumulate at the droplet interface, leading to the formation of a surface skin or shell. The timing of skin formation relative to internal moisture diffusion governs whether the resulting particles develop hollow, collapsed, or dense morphologies [24].

Single-droplet drying studies conducted under spray-drying-like temperature–time trajectories have demonstrated that formulation parameters, particularly the protein-to-lactose ratio, strongly influence particle morphology and surface composition. Using monodisperse MPC droplets generated by a pilot microfluidic spray dryer, Fang et al. [82] showed that increasing inlet temperature shifted particle morphology from spherical to deflated, increased protein denaturation, and reduced solubility. Importantly, the insoluble fraction was primarily casein rather than heat-labile whey proteins, supporting the view that casein-dominant surface or network formation can be central to rehydration failure in HPDPs. Similarly, Gaiani et al. [26] demonstrated that outlet air temperature altered surface composition and that surface lipid content correlated with wetting behavior. Together, these findings confirm that spray-drying conditions determine the interfacial structure governing both physical stability and functional recovery.

##### Thermal Trajectory

The thermal history of particles during spray drying is a critical determinant of structural evolution, particularly during the falling-rate period following shell formation. At this stage, particle temperature approaches the outlet air temperature as moisture content decreases [83]. If the particle surface temperature approaches or exceeds the effective Tg of the surface matrix, the surface enters a high-mobility state, increasing the probability of structural rearrangement, adhesion, and surface deformation during collision events [13,84].

This process should be interpreted as a kinetic consequence of a thermodynamic state [84,85]. A positive ΔT does not itself constitute stickiness or caking; rather, it increases molecular mobility and lowers viscosity, thereby accelerating time-dependent processes such as surface relaxation, viscous flow, adhesion, and wall deposition [79]. This distinction is particularly important for HPDPs, where protein-rich amorphous matrices may undergo structural rearrangement and interfacial reorganization even in the absence of lactose crystallization [79,86].

Droplet-to-particle formation frameworks emphasize that drying kinetics and particle solidification are strongly coupled and that the timing of surface skin formation plays a decisive role in determining final particle morphology, porosity, and internal stress distribution [11,24]. Early skin formation can trap internal moisture and lead to hollow or collapsed particles, whereas delayed solidification may result in denser structures with reduced porosity [12]. Both outcomes influence downstream stability and rehydration behavior by modifying water penetration pathways and inter-particle contact mechanics [28,87].

In milk-protein systems, thermal exposure and interfacial stresses during drying can induce protein structural changes, including denaturation and aggregation. Anandharamakrishnan et al. [88] and Haque et al. [89] showed that spray-drying variables, particularly inlet and outlet temperatures, significantly influence whey protein denaturation and the physicochemical properties of WPI powders. As discussed in Section 3.4, these structural changes are associated with reduced solubility, increased surface hydrophobicity, and impaired rehydration performance. Drying-induced protein transformations also modify the composition and functionality of the particle surface, governing stickiness during manufacturing and water penetration during reconstitution [22,26].

##### Outlet Relative Humidity

For dairy powders, outlet temperature alone does not uniquely determine final powder moisture content or *a_w_*. Instead, thermodynamic control frameworks identify outlet air RH as a key variable governing the equilibrium moisture. Since *a_w_* directly influences the Tg of amorphous matrices, outlet RH plays a central role in defining the initial mobility state of spray-dried particles [28,90,91].

Higher outlet RH corresponds to higher residual moisture content and *a_w_*, leading to Tg depression and a reduction in the glass transition margin. Under these conditions, particle surfaces may already be in a rubbery or near-rubbery state upon exiting the dryer, making them susceptible to adhesion, stickiness, and early-stage agglomeration. Conversely, maintaining lower outlet RH reduces *a_w_*, preserves a higher Tg relative to particle temperature, and limits surface plasticization and mobility-driven transformations [91].

This relationship is particularly critical in HPDPs, where instability is governed primarily by moisture-induced plasticization of protein-rich surface layers [19]. Even small increases in *a_w_* can enhance molecular mobility, promoting protein–protein interactions and interfacial restructuring that lead to cohesion and reduced rehydration performance [19,22]. Accordingly, controlling outlet RH is a key process parameter for minimizing the initial mobility state of HPDP particles and reducing the risk of immediate post-drying stickiness or later storage-induced consolidation.

### 4.2. Storage-Related Drivers of Instability in HPDPs

Storage-related instability in HPDPs is primarily governed by environmentally activated molecular mobility acting on a protein-dominant, structurally heterogeneous particle whose vulnerability has already been established during processing [92]. Temperature, RH, and *a_w_* define the thermodynamic state of the powder matrix, while the development of stickiness, uncontrolled agglomeration, caking, and rehydration loss occurs through kinetic processes that evolve over time. Therefore, storage instability should be understood as a time-dependent progression rather than an instantaneous response to a single threshold [92,93].

Across MPC, MPI, MCC, MCI, and high-grade WPC and WPI powders, storage commonly shifts powders along a continuum from surface softening to inter-particle adhesion, uncontrolled agglomeration, caking, surface densification, and persistent rehydration impairment. These outcomes are strongly amplified by humidity–temperature co-exposure, environmental cycling, and sustained consolidation load [18,54]. Case-study evidence in HPDPs links: (i) casein micelle surface restructuring and crust formation to solubility loss in high-protein MPC under warm and humid storage conditions; (ii) temperature–RH threshold behavior, including caking under severe combined conditions even in low-lactose whey and caseinate powders; and (iii) temperature-driven surface hydrophobicity evolution in WPI and β-lactoglobulin powders, particularly under elevated temperature or shipping-like cycling [14,15,16,54,70].

#### 4.2.1. Storage Temperature

Storage temperature is a primary driver of instability in HPDPs because it governs diffusion and relaxation kinetics, the viscosity of amorphous domains, and temperature-dependent sorption and redistribution processes within a metastable particulate matrix [13,14]. Relatively small increases in temperature can sharply accelerate instability once local domains enter a mobility-favorable state. This sensitivity is well established in dairy powder systems; ring-shear time-consolidation experiments in skim milk powder, used here as a mechanistic reference system rather than as a target HPDP, show a pronounced decrease in time-to-caking as storage temperature approaches Tg [18,54,94].

In HPDPs, temperature effects are most consequential when coupled with *a_w_* because moisture-induced plasticization can lower Tg into the storage-temperature range even when bulk moisture content appears low. In high-protein MPC powders, humidification and water uptake have been shown to significantly reduce Tg and thermomechanical strength, indicating that temperature safety margins are strongly dependent on humidity rather than fixed by composition alone [13,18,95].

Storage-driven solubility decline in high-protein MPC has been mechanistically linked to temperature-accelerated surface restructuring. Atomic force microscopy studies of MPC stored under elevated temperature and humidity show that solubility loss correlates with nanoscale surface features consistent with casein micelle restructuring and structural consolidation at the particle surface [54]. This indicates that temperature accelerates mobility-enabled surface densification and thereby impedes wetting and dispersion during rehydration [37,54].

A storage study on MPC powders spanning approximately 70–88% protein, stored for several weeks at 25 °C and 40 °C, demonstrated that higher storage temperature increased cohesiveness and reduced flowability. These results highlight that temperature influences not only physicochemical transformations but also inter-particle contact mechanics and time-dependent consolidation in powder beds [61].

For whey-protein-dominant powders, temperature can act as a regime-switch variable for surface modification and functional drift. Storage of WPI at elevated temperature and controlled *a_w_* has been associated with increased surface hydrophobicity and heterogeneity, indicating that temperature-driven surface evolution can impair wetting and dispersion even at relatively low *a_w_* [15,16].

Temperature cycling is particularly relevant under real storage and transport conditions, where repeated fluctuations can drive particles into and out of mobility-favorable states and increase the risk of condensation. Diurnal temperature variations in shipping containers can create transient local humidity increases and condensation events, thereby increasing *a_w_* and promoting caking under certain time–temperature scenarios [21,42]. These observations underscore the importance of considering transient temperature excursions in addition to steady-state storage conditions [95,96].

#### 4.2.2. Relative Humidity and Water Activity

Relative humidity and water activity are dominant storage drivers as they regulate plasticization of amorphous regions, capillary condensation, liquid bridge formation, and component redistribution at particle interfaces [13,14,18]. In thermodynamic terms, RH determines the moisture uptake tendency of the powder, while *a_w_* describes the equilibrium water status of the powder matrix. These variables define the mobility potential of the material. The observed instability outcomes, however, including stickiness, uncontrolled agglomeration, caking, and rehydration loss, are kinetic consequences that develop over time as moisture-enabled mobility promotes structural relaxation, inter-particle bonding, and consolidation [18,37,54].

As established in Section 3, moisture-induced Tg depression and the resulting increase in ΔT are primary activators of molecular mobility in protein-dominant HPDP matrices. However, Tg depression should be interpreted as an enabling state condition rather than direct evidence of caking. The development of caking requires subsequent time-dependent processes, including contact formation, liquid bridge growth, viscous flow, bridge strengthening, and mechanical consolidation.

Case-study evidence illustrates the sensitivity of HPDPs to moisture exposure. Maidannyk et al. [13] showed that controlled humidification of high-protein MPC powders markedly reduced Tg and thermomechanical strength, resulting in increased susceptibility to cohesion and reduced flowability. Fyfe et al. [17] demonstrated that exposure of MPC80 to elevated RH promotes surface restructuring and the formation of dense outer layers, which impede water penetration and contribute to reduced solubility and dispersibility. Burgain et al. [15] and Norwood et al. [16] showed that storage of WPI under controlled *a_w_* conditions leads to increased surface hydrophobicity and reduced wettability, confirming that moisture-driven surface evolution can impair rehydration even in the absence of visible caking.

RH fluctuations and cycling further exacerbate instability by repeatedly driving particles across mobility thresholds. These fluctuations alter the thermodynamic moisture state of the powder by changing local *a_w_* and Tg, but the resulting instability develops through time-dependent kinetic processes [18,19,21,42]. Adsorption–desorption cycles can induce structural relaxation, localized capillary condensation, and repeated formation and rupture of liquid bridges. This cyclic process promotes irreversible consolidation over time, even when average RH remains moderate [47,49]. In addition, humidity fluctuations can create spatially heterogeneous moisture distributions within powder beds, generating localized regions of elevated *a_w_* that act as initiation points for agglomeration and caking [95,96].

In practical storage and transport environments, humidity fluctuations are often coupled with temperature variations, increasing the risk of condensation and transient high-moisture exposure. Such events can rapidly plasticize particle surfaces, accelerate inter-particle bonding, and initiate instability processes that continue even after external conditions return to nominal levels [70,95,96].

#### 4.2.3. Capillary Condensation and Bridge Growth at High Relative Humidity

At sufficiently high RH, moisture can condense within inter-particle voids and form liquid bridges that sharply increase cohesion and promote caking. High RH and elevated *a_w_* describe the thermodynamic moisture state that enables capillary condensation, whereas bridge growth, viscous flow, inter-particle bonding, and strength development are kinetic consolidation processes that evolve over time. The rate and extent of this transition depend on particle contact geometry, packing density, surface composition, and mechanical load [13,14,42].

Phosanam et al. [14] conducted a factorial storage study of WPC35, WPI, sodium caseinate, and calcium caseinate at 25, 35, and 45 °C and 11, 44, and 85% RH, demonstrating that Tg decreased with increasing humidity across all temperatures. In lactose-containing matrices such as WPC35, lactose crystallization occurred under high humidity and was associated with caking. Importantly, caking was also observed under the most severe condition, 85% RH and 45 °C, in low-lactose whey and caseinate powders. This confirms that protein–protein interactions, moisture-induced plasticization, and minor-component redistribution, including surface fat migration, can drive caking in protein-dominant matrices independently of lactose crystallization.

Shipping-like oscillations in RH and temperature can further amplify instability by repeatedly inducing adsorption–desorption cycles and transitions between glassy and rubbery states. These cyclic environmental changes alter the thermodynamic moisture and mobility state of the powder, while the resulting physicochemical changes, including surface restructuring, protein modification, and functional deterioration, develop progressively over time [70,96]. Burgain et al. [70] showed that industrial WPC and β-lactoglobulin powders subjected to cyclic temperature and humidity profiles derived from real shipping conditions underwent progressive physicochemical changes, including protein modification, lactosylation, browning, and alterations in surface and functional properties. These findings demonstrate that fluctuating environmental conditions can accelerate structural and chemical evolution beyond what is observed under constant storage conditions.

**Figure 6:**
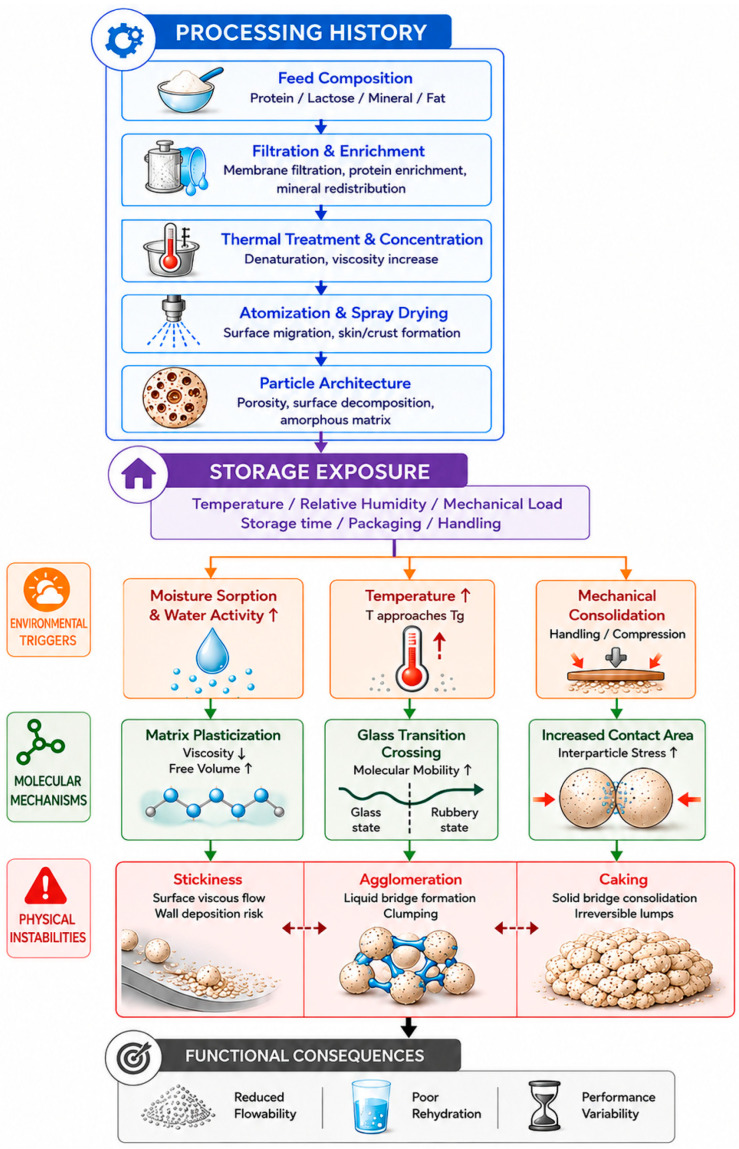
Integrated processing–storage–instability framework for HPDPs.

Figure 6 integrates the processing, storage, and rehydration domains within a single mechanistic framework. The framework illustrates the sequential and interconnected drivers of physical instability across three stages: (i) upstream processing, including membrane fractionation, concentration, and spray drying, which establishes particle architecture, surface composition, mineral balance, and the initial mobility landscape of the powder; (ii) storage, during which *a_w_* and temperature define the thermodynamic mobility state and activate time-dependent transformations, including stickiness, uncontrolled agglomeration, caking, and surface densification; and (iii) reconstitution, where cumulative structural evolution determines rehydration performance through its effects on wetting, dispersion, and dissolution. This framework emphasizes that instability in HPDPs is a continuous, mobility-driven progression rather than a series of isolated defects. The central role of Tg and *a_w_* as thermodynamic state descriptors linking processing, storage, and functionality is highlighted, while the resulting instability outcomes are presented as time-dependent kinetic processes that evolve over time.

#### 4.2.4. Packaging, Barrier Properties, and Consolidation Load

Packaging is a critical mediator of storage-related instability because it governs the rate of moisture ingress, the stability of internal headspace RH, and the likelihood of localized moisture redistribution during storage. Therefore, packaging influences the timescale over which water activity-driven plasticization and mobility-driven instability processes develop [21,41]. In this context, moisture-barrier performance should be considered an important storage-control factor for HPDPs because moisture uptake can promote surface plasticization, inter-particle bonding, and caking under unfavorable storage conditions [21,41,42].

In industrial storage, mechanical consolidation load arising from big bags, stacked sacks, and silo head pressure amplifies the effects of humidity and temperature by increasing inter-particle contact stresses [42]. Under elevated contact stress, particle surfaces plasticized by moisture sorption form cohesive micro-contacts more rapidly and at lower degrees of molecular mobility than unloaded powders [21,42]. Thus, consolidation load does not replace thermodynamic mobility criteria; rather, it accelerates the kinetic evolution from transient adhesion to mechanically resistant inter-particle bridging and caking once sufficient mobility has been achieved. Modeling work on milk-powder shipping suggests that, over typical transport durations, headspace temperature, humidity, and oxygen conditions may approach spatial uniformity within sealed bags, supporting the practical relevance of the bulk packaging environment to internal powder stability [60,96]. However, detailed studies linking packaging barrier properties, consolidation load, and time-dependent caking strength remain more common for milk powders broadly than for individual HPDP classes, representing an important gap in the literature for high-protein systems specifically [42].

Table 3 presents the impact of uncontrolled storage conditions on the development of physical instabilities in HPDPs.

## 5. Stability Enhancement Strategies for HPDPs

The stability of HPDPs must be addressed through a multi-scale approach encompassing molecular interactions, particle structure, processing conditions, and environmental control [18,19]. As discussed in previous sections, instability in HPDPs arises primarily from moisture-activated molecular mobility within protein-dominant amorphous matrices, leading to structural rearrangement, inter-particle bonding, and functional degradation [7,21]. Accordingly, modern stabilization strategies increasingly adopt an integrated ‘stability-by-design’ framework, in which formulation, processing, and storage conditions are simultaneously optimized to maintain a sufficient glass transition margin, minimize interfacial reactivity, and limit mobility-driven transformations [19,21]. Effective stabilization strategies can be broadly categorized into compositional design, process control and particle engineering, surface modification, environmental and packaging control, and predictive modeling. Importantly, these strategies must be interpreted in terms of their ability to control molecular mobility, inter-particle interactions, and structural accessibility, which collectively determine both physical stability and rehydration performance.

### 5.1. Compositional and Matrix Design

Compositional design is a primary tool for controlling the thermodynamic and kinetic stability of HPDPs. In systems containing residual lactose or other carbohydrates, increasing the proportion of high-Tg components or reducing hygroscopic constituents can elevate the composite glass transition temperature and reduce susceptibility to moisture-induced plasticization [19]. However, in high-protein matrices, compositional optimization must be carefully balanced, as excessive carbohydrate addition may reintroduce lactose crystallization pathways and associated caking mechanisms.

In HPDPs, particular emphasis is placed on mineral and ionic balance, which strongly influences protein–protein interactions and structural evolution [71,91]. Partial demineralization or calcium reduction in MPC and MCC systems reduces calcium-mediated casein–casein bridging and improves rehydration performance [3,34]. For example, calcium-reduced micellar casein concentrate shows improved dispersibility and functional properties relative to conventional micellar casein powders [34].

Similarly, Kommineni et al. [34] reported enhanced dispersibility in micellar casein powders with reduced calcium content, highlighting the role of mineral-mediated interactions in controlling functional behavior. Modification of protein composition also provides a means of reducing instability; incorporation of whey proteins or less aggregation-prone fractions can disrupt continuous casein networks and reduce the extent of protein association during storage [7,22,95]. While lipid content and emulsion structure may influence surface properties and wettability, their role in HPDP stability is generally secondary to protein and mineral interactions [7]. The use of stabilizing additives, such as hydrocolloids or polysaccharides, is limited in high-purity protein systems due to labeling constraints and potential effects on digestibility; nevertheless, in formulated systems, such additives may modify water binding, viscosity, and structural integrity, thereby influencing stability [7].

### 5.2. Process Control and Particle Engineering

Processing conditions, particularly during spray drying, play a decisive role in determining the initial structural state of HPDPs and their subsequent stability [18,19]. Control of drying parameters is essential to maximize the glass transition margin and minimize residual moisture, thereby reducing molecular mobility and susceptibility to structural evolution [18]. At the same time, excessive thermal exposure during drying can induce protein denaturation, exposing hydrophobic groups and sulfhydryl residues that promote aggregation during storage [89]. Studies have shown that controlling drying temperature and pre-heating conditions can preserve native protein structure and improve long-term stability and rehydration performance [82,89].

Particle engineering through controlled agglomeration (instantization) is widely used to improve wettability and dispersibility. Fluidized-bed agglomeration produces larger, porous granules that facilitate capillary-driven water penetration and enhance rehydration kinetics [33]. However, excessive porosity or insufficient structural strength may increase susceptibility to collapse or caking under humid conditions [33,97].

Upstream processing steps, including high-pressure homogenization, can further influence particle structure by modifying protein aggregation state and droplet size distribution prior to drying. These effects ultimately impact internal porosity, surface composition, and susceptibility to instability [12,24,26].

### 5.3. Surface Modification Techniques

The particle surface is the primary interface governing cohesion during storage and wetting during reconstitution. Surface modification strategies therefore focus on reducing hydrophobicity, controlling interfacial composition, and enhancing water accessibility [7,94]. Lecithination is one of the most widely used techniques, particularly for whey protein powders. Lecithin forms a hydrophilic coating that reduces the contact angle and improves wettability by facilitating initial water spreading and penetration [33]. Similar effects can be achieved through coating with hydrocolloids or carbohydrates, such as gum arabic or maltodextrin, which modify surface energy and delay moisture uptake [7].

Surface-active components may also act as barriers to oxygen and moisture, indirectly improving stability [7,98]. For example, the addition of antioxidants such as tocopherols can limit lipid oxidation and preserve surface properties in fat-containing matrices. More advanced approaches, including coacervation and nano-encapsulation, have been explored to create protective surface layers, although their industrial application remains limited [7]. Overall, effective surface modification must balance hydrophilicity, permeability, and structural stability, as excessive surface modification may alter functional properties or introduce new instability pathways [7,18,20].

**Figure 7:**
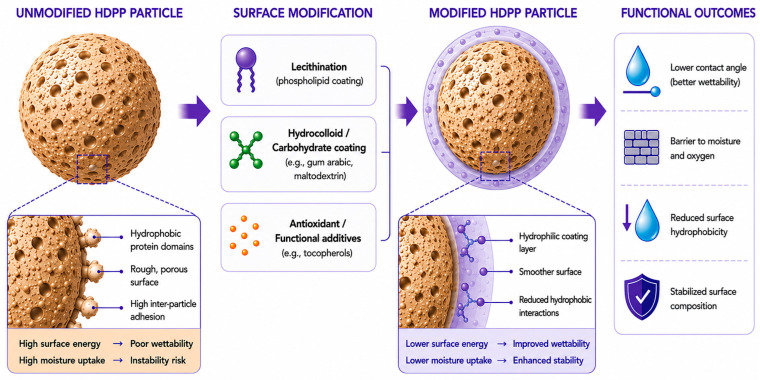
Schematic representation of surface modification strategies to enhance the physical stability and rehydration performance of HPDPs.

Figure 7 schematically represents potential surface modification strategies used to enhance the physical stability and rehydration performance of HPDPs. Surface treatments such as lecithination, hydrocolloid/carbohydrate coating, and antioxidant incorporation modify particle interfacial properties by reducing surface hydrophobicity, limiting moisture uptake, and decreasing inter-particle adhesion. The mechanisms, key benefits and examples of each surface modification method are given in Table 4.

### 5.4. Moisture and Packaging Control

Environmental control remains the most critical factor in preventing instability in HPDPs, as moisture-induced plasticization is the primary trigger of structural transformation. Packaging systems must therefore provide effective barriers to moisture and oxygen ingress, maintaining water activity below critical thresholds (typically *a_w_* < 0.2–0.3 for most HPDPs) [7,18]. High-barrier packaging materials, such as multilayer laminates (e.g., PET/Al/PE), are commonly used to minimize water vapor transmission rates [18]. In addition, desiccants and modified atmosphere packaging, such as nitrogen flushing, can further reduce internal humidity and oxidative reactions [7].

Storage temperature must also be controlled, as elevated temperatures reduce the effective glass transition margin and accelerate molecular mobility. Maintaining storage temperatures significantly below Tg (typically by 20–30 °C) is essential for long-term stability [19,20]. Thermal stability maps, combining Tg and water activity data, provide a useful framework for defining safe storage conditions. Mechanical factors, such as consolidation load during storage and transport, also contribute to caking by increasing inter-particle contact and accelerating bond formation. Proper handling practices, including controlled stacking and minimized compression, are therefore necessary to complement environmental controls.

### 5.5. Mineral and Ionic Optimization

Mineral composition plays a central role in governing protein interactions and structural evolution in HPDPs. Adjusting ionic strength and mineral equilibria can therefore be used to modulate stability and functionality [6,22]. For example, the addition of monovalent salts such as NaCl or KCl during diafiltration screens electrostatic interactions, displaces divalent calcium ions from the casein micelle surface, and substantially improves the solubility and rehydration of MPC80, with ionic-strength-modified powders achieving near-complete solubility compared to approximately 55% for untreated controls [55,101]. Controlled demineralization can similarly disrupt calcium-mediated bridging in casein-rich systems, improving dispersibility and reducing susceptibility to inter-particle consolidation during storage [34,63]. Chelating agents such as trisodium citrate, sodium phosphate, and sodium hexametaphosphate have also been used to sequester calcium ions, dissociate casein micelles, and improve MPC dissolution rate and solubility, although their use must be balanced against viscosity development, sensory, and regulatory considerations [102]. These strategies collectively highlight protein–mineral interactions as a critical and tractable control point in HPDP stability design.

### 5.6. Predictive Modeling and Stability-by-Design

Recent advances in predictive modeling have enabled the development of mobility-based stability frameworks that integrate thermodynamic, kinetic, and structural data [18,19,21,29]. These approaches combine sorption isotherms, glass transition models, such as the Gordon–Taylor equation, and kinetic concepts to define safe operating and storage conditions [18,19,21]. Dynamic mechanical analysis (DMA) can be used as a complementary technique to characterize mobility-related transitions and stickiness behavior in protein-containing dairy powder systems [31]. In high-protein powders, such approaches provide insight into the relationship between molecular mobility, thermomechanical behavior, and macroscopic properties such as caking and rehydration [13,14,37].

Recent advances in analytical characterization have expanded the ability to quantify molecular mobility and structural evolution in amorphous food powders. Techniques such as dynamic mechanical analysis (DMA), dielectric spectroscopy, and terahertz-based approaches provide complementary insights into relaxation phenomena occurring near the glass transition and offer promising opportunities for improving predictive stability models and stability-by-design frameworks [30,31,56].

A stability-by-design approach involves selecting compositions and processing conditions that maintain a sufficiently high Tg relative to expected processing and storage temperatures, followed by validation through accelerated storage studies under controlled temperature and humidity conditions [21,41]. Packaging and logistics conditions may then be incorporated into this framework to help maintain powders within acceptable stability domains throughout their lifecycle [21,41,42].

### 5.7. Developing Strategies Toward an Integrated Stability Framework

No single strategy is sufficient to ensure the stability of HPDPs. Instead, a holistic approach is required, integrating compositional design, processing optimization, particle engineering, and environmental control. Importantly, these strategies must be evaluated in terms of their combined effects on molecular mobility, interfacial properties, and structural accessibility. For example, increasing Tg through compositional modification may improve physical stability but simultaneously increase hygroscopicity, while demineralization may enhance rehydration at the expense of nutritional value. Therefore, stability optimization requires balancing competing effects across multiple scales. Recent advances in analytical techniques, including terahertz spectroscopy, dynamic mechanical analysis, and time-resolved scattering methods, provide new opportunities to directly monitor molecular mobility and structural evolution in HPDPs. By linking these measurements to functional outcomes such as flowability and solubility, it is possible to develop predictive design rules for next-generation dairy powders [13].

## 6. Research Gaps and Future Directions

Despite significant advances in understanding the physical instability of HPDPs, several critical knowledge gaps remain that limit the ability to predict and control their behavior under industrial and real-world conditions. While the role of moisture-activated molecular mobility is now well established, its quantitative relationship with structural evolution and functional degradation in protein-dominant powders remains insufficiently resolved.

A key research gap lies in the limited ability to directly link molecular-scale mobility with macroscopic properties such as caking strength, flowability, and rehydration performance. Although techniques such as DMA, dielectric spectroscopy, and terahertz spectroscopy have shown promise, their integration into predictive frameworks for industrial powders is still at an early stage. Further work is needed to establish robust correlations between mobility metrics (e.g., α-relaxation, sub-Tg transitions) and practical performance indicators. Future research should also integrate advanced mobility-sensitive analytical techniques, including dynamic mechanical analysis, dielectric spectroscopy, and terahertz spectroscopy, with predictive modeling approaches. Such integration could improve the mechanistic understanding of structural relaxation, moisture-induced plasticization, and instability development under realistic storage and transport conditions.

The interaction between processing history and storage behavior also requires deeper investigation. Although it is widely acknowledged that upstream processes such as membrane filtration and spray drying define the initial structural state, the extent to which specific processing parameters determine long-term stability remains difficult to predict. Comprehensive studies that combine controlled processing variations with long-term storage experiments are needed to establish causal relationships and define processing–stability maps. Real-world storage and transport conditions introduce additional complexity that is not adequately captured in most laboratory studies. Temperature and humidity fluctuations, mechanical loading, and packaging variability can lead to transient mobility states and localized instability, yet these factors are rarely incorporated into experimental designs. Future research should prioritize dynamic storage simulations and in situ monitoring approaches to better reflect industrial supply-chain conditions.

From a formulation perspective, the role of mineral composition, ionic strength, and protein association state requires further clarification, particularly in micellar casein-rich systems. While demineralization and ionic modification strategies have demonstrated clear functional benefits, their long-term effects on structural stability and nutritional quality remain incompletely understood. Finally, there is a need for integrated, multi-scale predictive models that combine thermodynamic, kinetic, and structural parameters. Current models based on glass transition and sorption behavior provide useful guidelines but often fail to capture the complexity of protein-dominant systems. The development of digital twins and data-driven approaches, supported by advanced analytical techniques, offers a promising pathway toward predictive stability-by-design frameworks. Addressing these gaps will be essential for advancing the rational design of HPDPs with improved stability, functionality, and robustness, particularly in the context of increasingly complex formulations and global distribution environments.

## 7. Conclusions

The physical stability of HPDPs is governed by a complex interplay between composition, structure, processing history, and storage environment, with moisture-activated molecular mobility emerging as the central unifying mechanism. In contrast to lactose-dominant systems, where instability is often driven by crystallization, HPDPs exhibit predominantly protein-mediated pathways of structural evolution, including surface plasticization, protein–protein association, and mineral-mediated consolidation. These processes link processing conditions directly to functional outcomes, such as flowability, caking behavior, and rehydration performance. This review demonstrates that instability in HPDPs is not the result of isolated phenomena but rather a continuous, mobility-driven progression initiated during processing and expressed under storage conditions. Spray drying plays a decisive role by establishing particle architecture, surface composition, and residual moisture, thereby defining the initial “mobility landscape” of the powder. Subsequent exposure to temperature and water activity governs the activation of instability mechanisms, highlighting the importance of controlling both processing-induced structure and environmental conditions. Effective stabilization therefore requires an integrated stability-by-design approach, in which compositional design, mineral and ionic balance, particle engineering, and process control are aligned with packaging and storage strategies. Among these, control of water activity and maintenance of a sufficient glass transition margin are critical to limiting molecular mobility and preventing structural rearrangement. At the same time, surface properties and particle architecture must be optimized to balance resistance to cohesion with accessibility during rehydration. Recent advances in analytical techniques and predictive modeling provide new opportunities to quantify molecular mobility and relate it to macroscopic powder behavior. These developments support the emergence of mechanistically grounded design frameworks capable of predicting instability and guiding formulation and process optimization. Future research should focus on refining these predictive approaches, particularly by integrating multi-scale structural characterization with real-world storage and transport conditions. Such efforts will be essential for developing next-generation HPDPs with improved stability, functionality, and robustness across increasingly demanding industrial and global supply-chain environments.

## Figures and Tables

**Table 1:** Dominant physical and functional instabilities and their associated mechanisms in HPDPs.

Continuum Stage	Operational Manifestation	Mechanism	Trigger	Reversibility/Outcome
Early cohesion	Stickiness	Surface relaxation; transient adhesive contact formation	Tg ↓/*a_w_* ↑	Partially reversible adhesion; initiation of inter-particle contacts
Intermediate aggregation	Uncontrolled agglomeration	Growth of inter-particle contacts into discrete clusters	*a_w_* ↑/ΔT > 0	Partly reversible to progressively irreversible clusters; reduced flowability
Advanced consolidation	Caking	Viscous sintering; capillary and solid bridge strengthening; protein- and mineral-mediated bonding	Time + ΔT > 0	Mechanically resistant mass; severe loss of flowability
Functional impairment	Rehydration loss	Restricted wetting; pore closure; clump formation; slow diffusion-controlled dissolution	Storage-induced structural evolution	Incomplete dispersion and dissolution; persistent sediment

↑: increase, ↓: decrease, Tg: glass transition, *a_w_*: water activity, ΔT: difference between storage temperature and glass transition temperature.

**Table 2:** Comparative classification of HPDPs based on origin, structural control, and dominant stability risks.

HPDP Class	Origin and Processing Route	Dominant Structural Control	Dominant Stability Risks Reported	Reference
MPC (transitional grades, e.g., MPC70)	Skim milk → UF/DF (casein: whey ~80:20) → moderate concentration → spray drying.	Mixed protein–lactose matrix; partial contribution of amorphous lactose phase; moderate protein–protein interactions	Processing: cohesive powder handling; potential sticking at unfavorable thermal/moisture conditions. Storage: moderate susceptibility to cohesion and rehydration decline compared with higher protein grades.	[7,22,40,60]
MPC (high-protein grades (MPC80–85)	Skim milk → UF/DF → high-protein retentate → spray drying	Protein-dominated amorphous matrix; contribution of strong protein–protein and protein–mineral interactions	Processing: poor bulk flowability; high cohesiveness at high protein content; increased dryer operational sensitivity. Storage: rehydration decline under elevated T/RH; caking/compaction; formation of lumps; persistent insoluble material on reconstitution.	[17,37,54,60,61]
MPI (≥90% protein)	Skim milk → UF/DF (casein:whey ~80:20) → extensive lactose/mineral removal → spray drying	Highly concentrated protein matrix; strong protein–protein interactions	Processing: cohesive fine powders; flowability limitations due to high protein content. Storage: rehydration impairment and persistent dispersibility limitations.	[25,33,40]
MCC (high-protein grades, e.g., MCC80–85)	Skim milk → MF (whey proteins removal; micellar casein retained) ± DF → spray drying	Mineral-stabilized casein network; CCP bridging; high structural density	Processing: high-viscosity concentrates; challenges in upstream handling and drying; cohesive powders post-drying. Storage: slow dispersion/rehydration; solubility sensitivity to mineral state; moisture-driven consolidation in contact zones.	[23,62,63]
MCI (≥90% casein)	Skim milk → MF with further purification/DF to very high casein levels → spray drying	Dense micellar packing; strong mineral-mediated interactions	Processing: cohesive powders; sensitivity to process history affecting micelle integrity. Storage: Increased consolidation via micelle–micelle interactions; moisture-driven densification.	[37,64]
WPC (high-protein grade, e.g., WPC80)	Cheese whey → UF/DF (protein enrichment) → spray drying	Protein–lactose matrix; residual lactose active; globular whey proteins at surface	Processing: stickiness due to lactose plasticization; drying sensitivity. Storage: caking and quality loss under adverse T/RH.	[15,28,65]
WPI (≥90% protein)	Cheese whey → UF/DF and/or ion exchange → spray drying	Protein-dominant; low lactose; surface-sensitive matrix	Processing: sensitivity to pre-drying heat history (aggregation state) and surface composition affecting wetting. Storage: surface hydrophobicity increases, impaired wettability, aggregation-driven insolubility.	[15,16,33]
Caseinates	Casein curd → neutralization to caseinate salt → drying	Non-micellar protein network; reduced mineral bridging; more open structure	Processing: stickiness risk. Storage: susceptibility to stickiness/relaxation in moisture-sensitive matrices; wetting limitations during reconstitution; fewer recent studies quantify “caking dominance” vs. micellar casein powders (flagged).	[28,66]

UF: ultrafiltration, DF: diafiltration, MF: microfiltration, T: temperature, RH: relative humidity, CCP: colloidal calcium phosphate, MPC: milk protein concentrate, MPI: milk protein isolate, MCC: micellar casein concentrate, MCI: micellar casein isolate, WPC: whey protein concentrate, WPI: whey protein isolate.

**Table 3:** The impact of uncontrolled storage conditions on the development of physical instabilities in HPDPs.

Improper Storage Condition	HPDP Studied	Dominant Observed Physical Instabilities	Mechanistic Interpretation Emphasized	Reference
Sustained warm and humid storage (warehouse hot spots; poor ventilation)	High-protein MPC (e.g., MPC80)	Strong solubility/rehydration decline; surface consolidation; formation of dense outer layers impeding water penetration	Mobility-enabled surface densification/micelle fusion impedes wetting and dispersion; protein-mediated inter-particle bridging	[17,54]
Elevated storage temperature (even at moderate RH)	MPC ~70–88% protein	Increased cohesiveness; poorer flow relative to cooler storage; progressive compaction	Time-dependent consolidation accelerated by temperature; contact evolution faster at higher temperature	[61]
High RH exposure (humidity abuse; unconditioned tropical storage)	MPC series (40–80%)	Tg strength decreases with humidification; increased susceptibility to sticking/caking; high-protein powders showed strong mobility shifts	Water plasticization lowers Tg/relaxation; increased molecular mobility and softening	[13]
High RH and high temperature combined (severe co-exposure)	WPC35, WPI, sodium caseinate, calcium caseinate	Caking at severe T/RH; Tg depression; loss of flowability; consolidation	At severe T/RH, mobility and/or capillary bridging drives cohesion even in low-lactose powders	[14]
High temperature at controlled low *a_w_*	WPI	Surface hydrophobicity increase; crackled surface; physicochemical drift	T-driven surface chemistry and restructuring at low *a_w_*; reduced wetting propensity	[15,16]
Realistic cycling (shipping/container cycling; day–night oscillations)	WPC and β-lactoglobulin powder (industrial grades)	Progressive surface property changes; altered wettability and reduced functional performance	Cycling drives repeated mobility excursions and cumulative surface evolution.	[70]
Long storage before use; mild-to-moderate abuse conditions	MCC/MCI	Decline in rehydration kinetics; surface densification; reduced dispersibility and dissolution rate	Storage-driven surface evolution/structural changes reduce rehydration kinetics	[23]
Moisture-induced mobility redistribution in mixed matrices	SMP vs. MPC50 vs. MPC80 vs. amorphous lactose	Distinct water states; differential caking behavior; lactose crystallization threshold behavior around *a_w_* changes	Mobility redistribution and crystallization can abruptly shift water binding and structural evolution	[47]
Packaging/condensation failures (leaky seals; container rain; poor headspace control)	Dry dairy products including casein/whey powders	Local wetting hotspots seed caking/agglomeration; episodic humidity shocks	Condensation and transient RH spikes create liquid bridges; desiccants/headspace management reduce risk	[42]
Packaging barrier tradeoff misspecification (using low-barrier monolayers for cost/sustainability without risk analysis)	Milk powder	Higher moisture ingress risk over shelf life; faster performance loss in humid climates	Barrier performance must be treated as a design variable in dry-food packaging selection	[96]

SMP: skim milk powder, T: temperature, RH: relative humidity, MPC: milk protein concentrate, WPC: whey protein concentrate, WPI: whey protein isolate, MCC: micellar casein concentrate, MCI: micellar casein isolate.

**Table 4:** The mechanism, key benefits, and examples of each surface modification method [21,36,70,96,99,100].

Approach	Mechanism	Key Benefits	Examples
Lecithination	Phospholipids orient at the interface, forming a hydrophilic outer layer that displaces hydrophobic groups.	Reduces the contact angle, improves dispersion and solubility, and enhances rehydration rate	Soy lecithin, sunflower lecithin
Hydrocolloid/Carbohydrate Coating	Forms a hydrophilic film that masks hydrophobic sites and limits moisture sorption.	Reduces surface hydrophobicity, retards moisture sorption, and improves flowability	Gum arabic, maltodextrin, pectin, pullulan
Antioxidant/Functional Additives	Reduce oxidative reactions and stabilize surface-active components against degradation.	Limits lipid oxidation, preserves surface functionality, and maintains color and flavor	Tocopherols, ascorbyl palmitate

## Data Availability

No new data were created or analyzed in this study. Data sharing is not applicable.
